# Human IL-21^+^IFN-γ^+^CD4^+^ T cells in nasal polyps are regulated by IL-12

**DOI:** 10.1038/srep12781

**Published:** 2015-08-04

**Authors:** Li Xiao, Lei Jia, Yannan Zhang, Sifei Yu, Xingmei Wu, Binyan Yang, Huabin Li, Changyou Wu

**Affiliations:** 1 Institute of Immunology, Zhongshan School of Medicine, Key Laboratory of Tropical Disease Control Research of Ministry of Education, Sun Yat-sen University, Guangzhou, PR China; 2 Allergy and Cancer Center, Otorhinolarygology Hospital, The First Affiliated Hospital of Sun Yat-sen University, Guangzhou, PR China

## Abstract

In the previous study, we found that the levels of IL-21 in nasal polyps (NPs) were significantly increased and associated with polyp size and recurrence. However, it is unclear that the cell source of IL-21 and the regulation of IL-21 in NP tissues. In the present study, we isolated the lymphocytes from NP tissues, uncinate tissues and peripheral blood of patients with NPs. The cells were analyzed for cell surface markers, cytokines and transcriptional factors by flow cytometry. The results indicated that CD4^+^ T cells were the major IL-21-exprssing cells in NP tissues and the majority of IL-21 producing CD4^+^ T cells co-expressed IFN-γ or IL-17A. IL-21^+^IFN-γ^+^CD4^+^ T cells in NP tissues exhibited the features of both Tfh and Th1 cells which co-expressed significantly higher amount of CXCR5, ICOS, PD-1, Bcl-6 and T-bet than did IL-21^+^IFN-γ^−^CD4^+^ T cells (p < 0.05). Treatment of the lymphocytes from NP tissues with IL-12 enhanced the production of IL-21 and IFN-γ, especially the frequency of IL-21^+^IFN^−^γ^+^CD4^+^ T cells (p < 0.05). The blockade of IL-12 inhibited the production of IL-21 and IFN-γ (p < 0.05). These findings indicated that IL-12 positively enhanced the generation of IL-21^+^IFN-γ^+^CD4^+^ T cells having the features of both Tfh and Th1 cells in NP tissues.

Nasal polyps (NPs) is a heterogeneous disease of upper airways characterized by persistent inflammation and repeated recurrence^1^. At present, the treatment outcomes of antibiotics, steroids and surgery for NPs are unsatisfactory and the recurrence rate remains high^2^. The etiology and pathogenesis of NPs^3^ is a matter of vigorous debate, but bacteria, viruses and fungi have all been implicated in the establishment of the inflammatory process. Studies in NPs have also convincingly shown that the pathologic process consists of an aberrant immune-inflammatory response. Some evidence demonstrates that^4^,^5^,^6^ T helper (Th) cells, especial Th17, Treg or Th2, are important mediators of the pathologic response in the NPs microenvironment. T cell-derived cytokines^7^,^8^, such as IFN-γ, IL-4, TNF-α, IL-17 and IL-10, have been proved to implicate in regulating the inflammatory responses of the nasal sinus.

IL-21, a member of the common-γ chain (γc) family of cytokines, has ability to act on multiple cells of the immune system. Several studies have indicated that^9^ IL-21 regulates the differentiation, growth and activation of CD4^+^, CD8^+^ T cells as well as NK cells, whereas myeloid cells, including dendritic cells and macrophages, are also stimulated by IL-21. Consistent with these broad affects, it has become clear that^10^ not only does IL-21 regulate normal lymphoid development and function, but it also serves critical roles in inflammatory, allergic, autoimmune and tumorous diseases. For instance^11^, in mucosal inflammation of gut there is enhanced production of IL-21 that regulates the production of Th1-associated cytokines and the balance between Treg and Th17 cells. And neutralization of IL-21 could be a valuable addition to the therapeutic method to combat inflammatory diseases.

In previous studies^12^, we found that the levels of IL-21 were significantly increased in NP tissues than in uncinate tissues. Moreover, IL-21 promoted the differentiation of plasma cells and the production of Igs and was positively related to polyp size and recurrence after surgery. However, the source of IL-21 in NP tissues has not been expatiated. In addition, the characteristic of IL-21-expressing cells and the basic mechanisms that control IL-21 expression in NP tissues are not clear. In this study, we performed a signal-cell analysis of IL-21-producing T cells to ascertain which cells produce IL-21 in NP tissues and found that CD4^+^ T cells were the major source of IL-21 producing cells which are Tfh-like cells. In addition, we investigated the factors involved in the regulation of Tfh cells or Tfh-like cells generation in NP tissues.

## Result

### IL-21 was produced and expressed mainly by CD4^+^ T cells in human NP tissues

**Figure 1:**
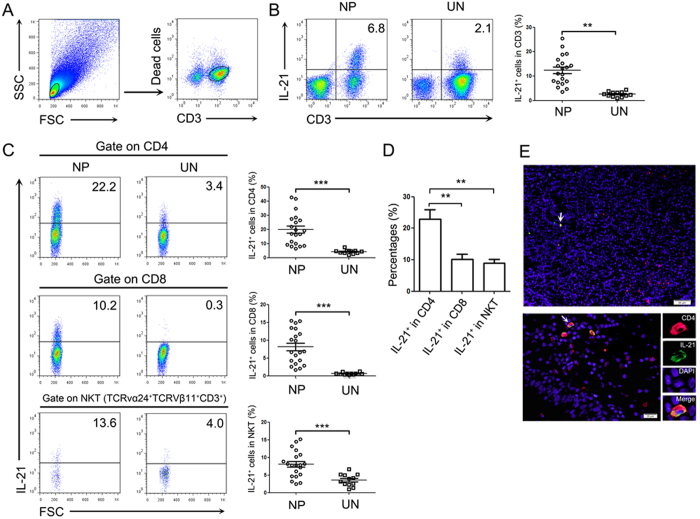
IL-21 was produced and expressed mainly by CD4^+^ T cells in NP tissues. (**A**) FACS gating was used in the analysis of lymphocytes in NP tissues and uncinate tissues. (**B**) Representative FACS data and statistical analysis showed the expression of IL-21 in lymphocytes (NP: n = 20; UN: n = 12). (**C**) Representative FACS data and statistical analysis showed IL-21 secretion from CD4^+^ T cells, CD8^+^ T cells and Vα24^+^ T (NKT) cells (NP: n = 20; UN: n = 12). (**D**) Summary data showed the percentages of IL-21^+^ cells in the CD4^+^ T cells, CD8^+^ T cells, and TCRvα24^+^ T (NKT) cells from NP tissues (n = 15). (**E**) Representative microphotographs of CD4 (red), IL-21 (green) and DAPI (blue)-stained paraffin sections of NP tissues. The arrows display the colocalization of CD4 and IL-21. Scale bars represent 50 Μm or 20 μM. Data were shown as mean ± SEM. Statistical significance was determined with the Mann–Whitney test. **p < 0.01; ***p < 0.001. NP, nasal polyps; UN, uncinate.

Interleukin-21 is a cytokine that has broad effects on both innate and adaptive immune responses. In previous study, we found that there were increased levels of IL-21 in NP tissues than uncinate tissues. To investigate the source of IL-21, we performed a single cell analysis by FACS using lymphocytes isolated from NP tissues and uncinate tissues. We found that the major IL-21-producing cells were CD3^+^ T cells (Fig. 1A,B). The fraction of IL-21-producing CD3^+^ T cells was significantly higher in NP tissues compared with uncinate tissues. Furthermore, IL-21 was expressed by CD4^+^ T cells, CD8^+^ T cells and TCRvα24^+^TCR_V_β11^+^ (NKT) cells. The percentage of IL-21 in CD4^+^ T cells, CD8^+^ T cells and NKT cells were substantially increased in NP tissues compared with uncinate tissues (Fig. 1C). Among all IL-21-producing cells, the percentages of IL-21 in CD4^+^ T cells were much higher than in CD8^+^ T cells and NKT cells (Fig. 1D). CD4^+^IL-21^+^ T cells were readily seen in NP tissues as revealed by immunofluorescent staining (Fig. 1E). These results indicated that CD4^+^ T cells were the predominant IL-21-producing cells in human NP tissues.

### The majority of IL-21^+^CD4^+^ T cells co-expressed IFN-γ in NP tissues

**Figure 2:**
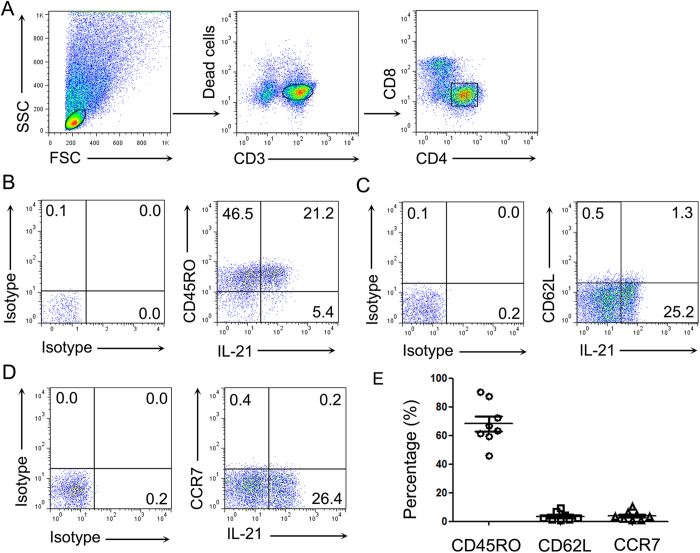
The majority of IL-21^+^CD4^+^ T cells in NP tissues were memory effector cells. (**A**) FACS gating was used in the analysis of lymphocytes in NP tissues. (**B–D**) Representative flow cytometric graphs showed the expression of CD45RO, CD62L or CCR7 on CD4^+^ T cells in NP tissues. (**E**) Summary data showed percentage of CD45RO^+^ cells, CD62L^+^ cells, and CCR7^+^ cells in IL-21^+^CD4^+^ T cells from NP tissues (n = 8). Data were shown as mean ± SEM.

The analysis of phenotypes for lymphocytes showed that the expressions of CD45RO, CD62L and CCR7 on CD4^+^ T cells in NP tissues were increased compared to those of uncinate tissues (Supplementary Figure1). We next examined the characteristics of IL-21-expressing CD4^+^ T cells in human NP tissues. We found that approximately 80% ~ 90% of IL-21^+^CD4^+^ T cells in NP tissues expressed CD45RO, but rarely expressed CD62L and CCR7 (Fig. 2). These results indicated that the majority of IL-21^+^CD4^+^ T cells in NP tissues were effector memory cells.

**Figure 3:**
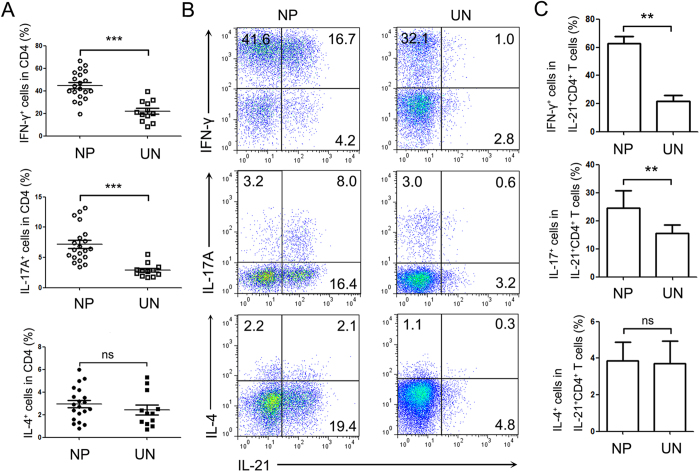
The majority of IL-21^+^CD4^+^ T cells in NP tissues co-expressed IFN-γ. (**A**) The statistical results showed the percentages of IFN-γ, IL-17A and IL-4 expression in CD4^+^ T cells (NP: n = 20; UN: n = 12). (**B**) Representative flow cytometric analysis of IFN-γ, IL-17A, IL-4 and IL-21 expression by CD4^+^ T cells was shown. (**C**) Statistical results showed that the percentages of IL-21^+^ cells co-expressed IFN-γ, IL-17A and IL-4 in CD4^+^ T cells (n = 12). Data were shown as mean ± SEM. Statistical significance was determined with the Mann–Whitney test. **p < 0.01, ***p <0.001; ns: no significance. NP, nasal polyps; UN, uncinate.

Although it is recognized that IL-21 is mostly produced by Th17, Tfh and NKT cells in human systems, other additional T cell subsets also secret IL-21. There we determined whether IL-21-producing cells co-expressed Th1-, Th2- or Th17-related cytokines in NP tissues. FACS analysis indicated that there were higher percentages of IFN-γ- and IL-17A-producing cells in CD4^+^ T cells from NP tissues than uncinate tissues and the expression levels of IL-4 in CD4^+^ T cells were no significantly changed between two groups (Fig. 3A). The further studies showed that the majority of IL-21-producing CD4^+^ T cells co-expressed IFN-γ (Th1 cells), but not IL-17A (Th17 cells) and IL-4 (Th2 cells) (Fig. 3B). And the ratios of IFN-γ^+^ cells and IL-17A^+^ cells in IL-21^+^CD4^+^ T cells were significantly higher in NP tissues than in uncinate tissues (Fig. 3C). These data suggested that most of IL-21^+^CD4^+^ T cells were IFN-γ producing cells in NP tissues.

### The expression of CXCR5, PD-1 and ICOS on CD4^+^ T cells in NP tissues was significantly increased

**Figure 4:**
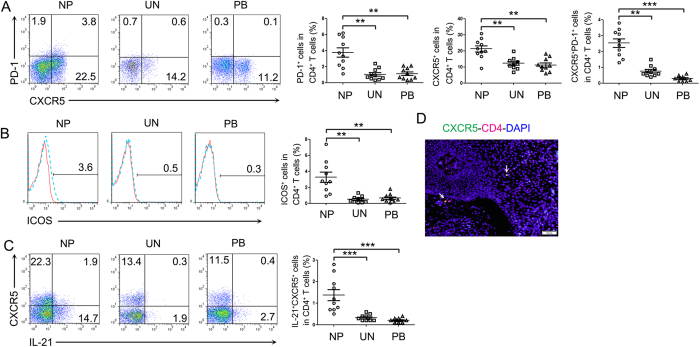
The expression of CXCR5, PD-1 and ICOS on CD4^+^ T cells in NP tissues was significantly increased. (**A,B**) Representative FACS data and statistical results showed the expression of CXCR5, PD-1 and ICOS of CD4^+^ T cells in the lymphocytes of NP tissues, uncinate tissues and PBMCs (n = 10). (**C**) Representative FACS data and statistical results showed the expression of CXCR5 on CD4^+^IL-21^+^ cells (n = 10). (**D**) Representative microphotographs of CD4 (red), CXCR5 (green) and DAPI (blue)-stained section of NP tissues. Arrow indicated CXCR5-expressing CD4^+^ T cells (yellow). Scale bars represent 50 Μm. Data were shown as mean ± SEM. Statistical significance was determined with the Mann–Whitney test. **p < 0.01; ***p < 0.001. NP, nasal polyps; UN, uncinate; PB, PBMCs.

In human circulating systems, CXCR5^+^CD4^+^ T cells are thought to have some features of Tfh cells and are involved in the development of immune-mediated diseases. We detected the expression of basic phenotypes related to Tfh cells on CD4^+^ T cells. The data showed that CXCR5 expressing CD4^+^ T cells existed in NP tissues, PBMCs and uncinate tissues. The expression of CXCR5, PD-1 and ICOS on CD4^+^ T cells in NP tissues was significantly increased compared to PBMCs and uncinate tissues (Fig. 4A,B). Immunofluorescence of parafin sections showed the cells positive for both CD4 and CXCR5 (Fig. 4D). Further studies indicated that some of IL-21-expressing CD4^+^ cells were positive for CXCR5. The percentages of IL-21–producing CXCR5^+^ cells in CD4^+^ T cells were higher in NP tissues than in PBMCs and uncinate tissues (Fig. 4C).

### IFN-γ^+^IL-21^+^CD4^+^ cells in NP tissues have the characteristics of both Tfh cells and Th1 cells

**Figure 5:**
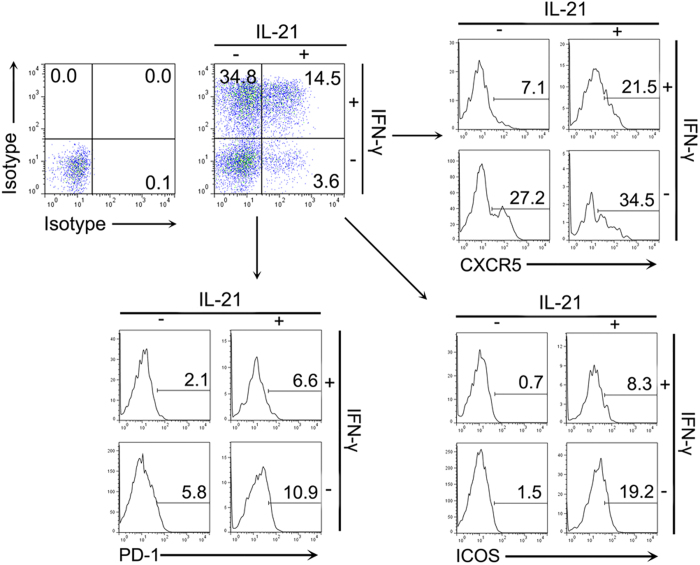
IFN-γ^+^IL-21^+^CD4^+^ T cells co-expressed CXCR5, PD-1, ICOS. CD4^+^ cells were gated in lymphocytes from NP tissues and the production of IL-21 and IFN-γ were detected. The expression of CXCR5, PD-1 and ICOS in each cell subset was analyzed. Representative data of five independent experiments were shown.

We next examined whether IL-21^+^IFN-γ^+^CD4^+^ T cells in NP tissues have the characteristics of Tfh cells and Th1 cells. To evaluate the characteristics of cell subsets, CD4^+^ T cells were divided into four subpopulation: IFN-γ^+^, IL-21^+^, IFN-γ^+^IL-21^+^ and IFN-γ^−^IL-21^−^ T cells, according to the production of IL-21 and IFN-γ. The expression of CXCR5, PD-1 and ICOS on these cell subsets was analyzed. The results indicated that IFN-γ^+^IL-21^+^ T cells expressed higher levels of CXCR5, PD-1 and ICOS compared to IFN-γ^+^IL-21^−^ T cells in CD4^+^ T cells of NP tissues (Fig. 5).

**Figure 6:**
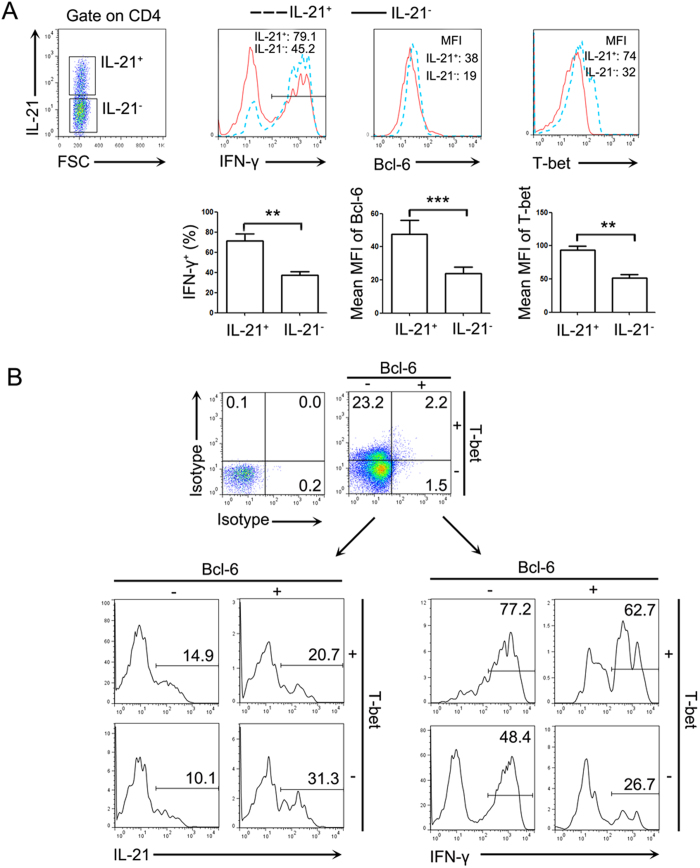
Some of IFN-γ^+^IL-21^+^CD4^+^ cells co-expressed transcription factors of Bcl6 and T-bet. (**A**) IL-21^+^ and IL-21^−^ cells were gated on CD4^+^ T cells in lymphocytes from NP tissues. The expression of IFN-γ, Bcl6 and T-bet were analyzed (n = 6). Data were shown as mean ± SEM. Statistical significance was determined with the Mann–Whitney test. *p < 0.05 (**B**) The CD4^+^ T cells were gated according to the expression of Bcl6 and T-bet in NP tissues. The expression of IFN-γ and IL-21 in each cell subset was analyzed. Data were representative of five separate experiments with similar results.

Because the contribution of transcription factors to cytokines expression, we examined the expression of Bcl6 (for IL-21) and T-bet (for IFN-γ) in CD4^+^ T cells of NP tissues. The lymphocytes isolated from NP tissues were stimulated with PMA plus ionomycin and BFA for 5 h, and detected by FACS. The expression levels of transcription factors did not change with this short stimulation (data not shown). However, IL-21^+^ cells expressed significantly higher amount of IFN-γ, Bcl-6 and T-bet compared to IL-21^−^ cells in CD4^+^ T cells (Fig. 6A).

To determine the regulation of transcription factors to CD4^+^ T cells co-expressed cytokines in NP tissues, CD4^+^ T cells were gated on the basis of expression of Bcl-6 and T-bet. The expression of IL-21 and IFN-γ by each cell subsets was analyzed. Compared to isotype staining, Bcl6^+^T-bet^+^ cells expressed higher percentage of IFN-γ than Bcl-6^+^T-bet^−^ cells, and expressed higher percentages of IL-21 than Bcl-6^−^T-bet^+^ cells (Fig. 6B). These results indicated that some of CD4^+^ T cells in NP tissues co-expressing IL-21 and IFN-γ were regulated by Bcl-6 and T-bet together, and CD4^+^IL-21^+^IFN-γ^+^ T cells had the characteristics of Tfh cells and Th1 cells in NP tissues.

### IL-12 enhances IL-21 and IFN-γ production in CD4^+^ T cells in NP tissues

**Figure 7:**
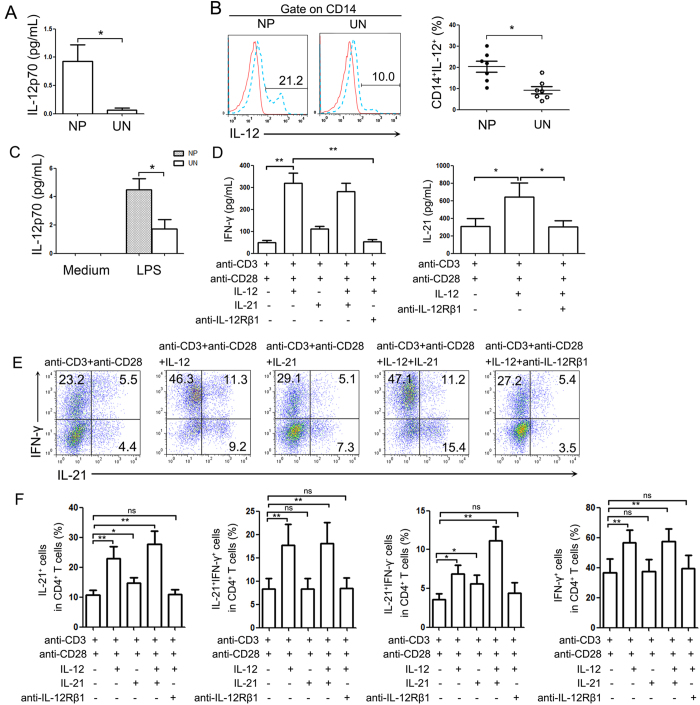
IL-12 enhances the production of IL-21 and IFN-γ by CD4^+^ T cells. (**A**) The concentrations of IL-12p70 in the homogenates of NP and uncinate tissues were detected with ELISA (n = 8). (**B**) The lymphocytes from NP tissues and uncinate tissues were stimulated with LPS for 8 h and the expression of IL-12 by CD14^+^ cells was assayed by FACS (n = 7). (**C**) The lymphocytes from NP and uncinate tissues were stimulated with LPS for 24 h and the levels of IL-12p70 were detected with ELISA (n = 7). (**D**) The lymphocytes isolated from NP tissues were stimulated for 3 days with anti-CD3 and anti-CD28 in the presence or absence of IL-12 or IL-21 and anti-IL-12Rβ1 antibodies. The statistical results showed the concentration of IL-21 and IFN-γ in cell-free supernatants. (**E**) The stimulated condition was the same as (**D**), PMA and ionomycin plus BFA were added in the last 5 hours. The representative data and statistical results of five independent experiments showed the expression of IL-21 and IFN-γ in CD4^+^ T cells. Data were shown as mean ± SEM. Statistical significance was determined with the Mann–Whitney test. *p < 0.05; **p < 0.01, ns: no significance. NP, nasal polyps; UN, uncinate.

The concentrations of IL-12p70 were increased in homogenates of NP tissues than in uncinate tissues (Fig. 7A). The cells from NP tissues produced higher levels of IL-12 compared to uncinate tissues after stimulation with LPS (Fig. 7B,C). The lymphocytes isolated from NP tissues were stimulated with IL-12 (or IL-21) in the presence or absence of anti-IL-12Rβ1 mAb for 3 days. The concentrations of IL-21 and IFN-γ in cell culture supernatants were increased after the stimulation with IL-12 (Fig. 7D). And the further analysis of cells showed that the addition of exogenous IL-12 significantly increased the percentages of IL-21 and IFN-γ double positive CD4^+^ T cells (Fig. 7E,F). IL-21 also increased the percentages of IL-21^+^CD4^+^ T cells. However, IL-12 induced not only the percentage of IL-21- or IFN-γ-producing cells, but also the fraction of cells co-expressing both cytokines. The generation of IL-21 and IFN-γ after blocking the effect of IL-12 with anti-IL-12Rβ1 mAb was inhibited.

## Discussion

Nasal polyps is a chronic inflammation disease of the nasal cavity and sinus. It has been shown that eosinophils and lymphocytes are the most common inflammatory cells in NP tissues^13^. T lymphocytes are the center to the development of sinonasal inflammation. The initiation and regulation of nasal inflammatory responses are dependent on T lymphocyte recruitment, activation and cytokine secretion^14^. IL-21 is a cytokine that has multiple roles in regulating both innate and adaptive immune responses. It is proved that IL-21 has the potential to impact many aspects of the immune-mediated inflammatory disorders^15^, for inflammatory bowel diseases, systemic lupus erythematosus, diabetes and rheumatoid arthritis. In allergic rhinitis of mouse mode^16^, intranasal administration of rmIL-21 effectively ameliorated allergic symptoms and suppressed the production of allergen-specific IgE. In the previous study^12^, our work demonstrated that the concentrations of IL-21 were significantly higher in NP tissues than in normal uncinate tissues. However, it is still not clear that which cells produces IL-21 and how IL-21 production is regulated in NP tissues.

Although it is recognized that IL-21 is produced by activated CD4^+^ T cells, NKT cells and Th17 cells. There is evidence that other Th subsets can synthesize this cytokine under specific circumstances. In mice, IL-21 is preferentially produced by Th17 and Th2 cells, but not Th1 cells^17^. In contrast, studies in human show that Th17 cells produce higher levels of IL-21 than Th2 cells, and that IL-21 functions as an autocrine growth factor for Th17 cells^18^,^19^. Additionally, Th1 cells also produce IL-21 in some inflammatory condition including inflammatory bowel disease (IBD)^20^. In this study, we demonstrated that IL-21 is almost exclusively secreted by CD3^+^ cells and CD4^+^ T cells are the major producer of IL-21 in NP tissues by flow cytometric analysis. The further studies showed that the majority of IL-21-producing CD4^+^ T cells were terminally differentiated effector memory cells in NP tissues, displaying CD45RO^+^CCR7^−^CD62L^−^ phenotype. Most of IL-21-producing CD4^+^ T cells in NP tissues co-express IFN-γ, to a lesser extent IL-17A or IL-4. And the percentages of co-expressing IFN-γ cells in IL-21^+^CD4^+^ T cells were significantly higher in NP tissues than uncinate tissues, suggesting that increased polyfunctional CD4^+^ T cells existed in NP tissues.

IL-21 is also produced by T follicular helper (Tfh) cells^21^, a newly defined subpopulation of Th cells in human tonsils. Distinguishing features of Tfh cells^22^ are the expression of CXCR5, PD-1, SAP, IL-21, ICOS and other molecules. In this study, we analyzed the basic characteristic markers of Tfh cells on CD4^+^ T cells in NP tissues with those of PBMCs and uncinate tissues. The surface marker analysis showed that the percentages of CXCR5, PD-1, ICOS on CD4^+^ T cells were increased in NP tissues than in uncinate tissues and PBMCs. Some of CXCR5^+^ T cells were IL-21-producing CD4^+^ T cells. The fraction of IL-21-producing CXCR5^+^CD4^+^ T cells was higher in NP tissues than in PBMCs and uncinate tissues. These data suggested that the expression of CXCR5, PD-1 and ICOS on CD4^+^ T cells was higher in NP tissues than in uncinate tissues.

In the past decade, there have been important advances in our knowledge and understanding Tfh cells. Except “conventional” Tfh cells, “unconventional” subsets of Tfh cells have also been identified including extrafollicular Th cells, NKT follicular helper (NKTfh) cells, γδTfh cells, follicular Treg cells and circulating Tfh (CD4^+^CXCR5^+^) cells^23^. Though there are clear differences between CD4^+^CXCR5^+^ T cells in the blood and in tonsils, human circulating Tfh (or Tfh-like) cells actually have some features of Tfh cells^24^. Recently, there is growing studies concerning the involvement of circulating Tfh-like cells in chronic inflammatory diseases^25^,^26^, for HIV, chronic hepatitis B, lupus and rheumatoid arthritis. Moreover, Tfh cells are preferentially thought only in lymphatic organ, but not solid tissues. However, the recent study firstly found^27^ CXCL13-producing CD4^+^ Tfh cells in breast cancer which predicted survival or preoperative response to chemotherapy. However, it is a problem that whether there are Tfh or Tfh-like cells in NP tissues.

In NP tissues, we found the majority of IL-21^+^CD4^+^ T cells co-expressed IFN-γ. The studies about the phenotype characteristics showed that these IL-21^+^IFN-γ^+^CD4^+^ T cells highly expressed CXCR5, PD-1, ICOS compared to IL-21^−^IFN-γ^+^CD4^+^ T cells. Because the regulation of transcription factors to Tfh cells, we observed the expression levels of Bcl-6 and T-bet in IL-21^+^CD4^+^ T cells. The results showed that IL-21^+^CD4^+^ T cells expressed higher amount of IFN-γ, Bcl-6 and T-bet than did IL-21^−^CD4^+^ T cells in NP tissues. And IL-21^+^IFN-γ^+^CD4^+^ T cells co-expressed Bcl-6 and T-bet compared to IL-21^−^IFN-γ^+^CD4^+^ T cells in NP tissues. Taken together, these data suggested that some of IL-21^+^CD4^+^ T cells in NP tissues were called Tfh-like cells which expressed IFN-γ and had fundamental characteristics of Tfh cells. Many research groups demonstrated that B cells might play an important role in the inflammatory response in nasal polyps. There are increased BAFF or CXCL-13 protein level^28^, the numbers of B cells, plasma cells^29^ and even ectopic B-cell follicles^30^ in NP tissues than in control nasal mucosa. We suggested that the gathering and differentiation of B cells may be related with the function of Tfh-like cells in NP tissues.

In murine studies^31^, IL-12 induced the expression of both T-bet and Bcl-6 genes in early stage of Th1 cell differentiation, produced IL-21- and IFN-γ producing cells which share features of both Tfh and Th1 cells. In human studies^32^, there are similar experimental results. IL-12 produced by activated dendritic cells induced naïve CD4^+^ T cells to become different IL-21- producing Tfh-like cells: IL-21^+^IFN-γ^+^T-bet^+^ cells and IL-21^+^IFN-γ^−^T-bet^−^ cells. Previous reports and our results demonstrated that IL-12 was increased in NP tissues^33^,^34^. *In vitro* studies demonstrated that IL-12 increased the production of IL-21 and IFN-γ of CD4^+^ T cells in NP tissues, especially the genetation of Tfh-like cells that expressed both IL-21 and IFN-γ.

Taken together, our studies suggested that there were increased Tfh-like cells in NP tissues than in uncinate tissues, which had the characteristics of Tfh cells and Th1 cells. IL-12 enhances IL-21 and IFN-γ production in CD4^+^ T cells in nasal polyps, especially the percentages of Tfh-like cells. Some evidences^35^ indicate that IFN-γ^+^IL-17^+^ cells are potentially pathogenic Th17 cells in multiple sclerosis patients and reduction of IFN-γ-producing Th17 cell numbers is a potential strategy for the treatment of autoimmune disease. IL-21 promoted the differentiation of Th17 cells but inhibited the generation of pathogenic Th1/Th17 cells^36^. Therefore, the function of these Tfh-like cells in NP disease is needed to be further studied.

## Materials and Methods

The study has been approved by Zhongshan School of Medicine and the First Affiliated Hospital of Sun Yat-sen University. All methods used in this study were carried out in accordance with the approved guidelines and all experimental protocols were approved by Institute of Immunology, Zhongshan School of Medicine of Sun Yat-sen University.

### Patients

A total of 42 subjects (42 patients with NPs; 19 control subjects) were recruited from the First Affiliated Hospital of Yat-sen University. NP tissues were obtained during routine functional endoscopic sinus surgery. The diagnosis was made according to the criteria of the European Position Paper on Rhinosinusitis and Nasal Polyps 2007 (EP3OS 2007). The control group comprised patients undergoing septoplasty for anatomic variations, uncinate tissues were removed during septal surgery. Patients with an established immunodeficiency, pregnancy, diagnosis of classic allergic fungal sinusitis or cystic fibrosis were excluded from the study. Details of subjects’ characteristics see Table 1. None of the subjects used oral or intranasal steroids for at least 2 weeks before sample collection. Peripheral blood samples from patients with NP were collected.

All subjects signed informed consent forms and the study was approved by the Ethical Committee of the First Affiliated Hospital and Zhongshan School of Medicine of Sun Yat-sen University.

### Cell isolation and tissue homogenate

Tissue samples were cutted into small pieces, digested in RPMI-1640 medium with endotoxin-free collagenase I (2 mg/ml, Sigma-Aldrich, St Louis, MO, USA) for 1 h at 37 °C. The digested tissues were filtered through a 100-mm cell nylon mesh (BD Bioscience PharMingen, San Diego, CA, USA) to prepare a single cell suspension. The lymphocytes in NP tissues and uncinate tissues were obtained by Ficoll-Hypaque (Tianjin Hao Yang Biological Manufacture, Tianjin, China) density gradient centrifugation. PBMCs were isolated by Ficoll density gradient centrifugation from peripheral blood of patients with NP.

Freshly tissue specimens were weighed, supplemented with PBS (1 mL of PBS/100 mg of tissue) containing a protease inhibitor cocktail (Keygentec, Nanjing, Jiangsu, China), and homogenized on ice for 1 minute. The suspension was then centrifuged at 4000 rpm for 20 minutes at 4 °C. The supernatants were stored at −80 °C for ELISA.

### Flow cytometry

Cells were incubated in red fluorescent reactive dye (live/dead fixable dead cell stain kits, Invitrogen) for 30 minutes for dead cell discrimination. The cells were washed twice with PBS buffer containing 0.1% BSA and 0.05% sodium azide. For surface stainings, cells were incubated with the respective mAbs at 4 °C in the dark for 30 min. For the detection of intracellular cytokines, cells were fixed with 4% paraformaldehyde and permeabilized in PBS buffer containing 0.1% saponin (Sigma-Aldrich), 0.1% BSA and 0.05% NaN_3_ for at least 2 h or overnight at 4 °C and stained with conjugated mAbs for intracellular cytokines. For the detection of intracellular transcription factors, cells were stained for surface antigens, followed by fixation, permeabilization with Permeabilization/Fixation buffer (BD Bioscience PharMingen) and staining according to the protocol of Permeabilization/Fixation Kit. Stained cells were washed twice before analysis using BD FACS Aria II (San Jose, CA, USA). Lymphocytes were gated on forward and side scatter profiles and analyzed using FlowJo software (Treestar, San Carlos, CA, USA).

The following mAbs were used for cell surface or intracellular stainings: FITC-labeled anti-CD45RO (clone: UCHL1), PE-labeled anti-IL-17 (clone: N49-653), anti-IL-12p40/p70 (clone: C11.5), anti-IL-21 (clone: 3A3-N2.1), anti-T-bet (clone: 4B10), anti-PD-1 (clone: MIH4), APC-Cy7-labeled anti-CD4 (clone: GK1.5), APC-labeled anti-IFN-γ (clone: B27), anti-IL-4 (clone: 8D4-8), anti-CD62L (clone: DREG-56), anti-ICOS (clone: ISA-3), PE-cy7-conjugated anti-CD8 (clone: RPA-T8), anti-CCR7 (clone:3D12), anti-CD14 (clone: M5E2), Alexa Fluor488-labeled anti-CXCR5 (clone: RF8B2), Alexa Fluor647-labeled anti-CXCR5 (clone: RF8B2), anti-IL-21 (clone: 3A3-N2.1), PE-CF594-labeled anti-Bcl6 (clone: K112-91), anti-CD3 (clone: SP34-2), isotype-matched control antibodies, purified anti-CD3 and anti-CD28 mAb were purchased from BD Bioscience PharMingen. PE-labeled anti-TCRVα24 (clone: C15) and FITC-labeled anti-TCRVβ11 (clone: C21) were purchased from Beckman-Coulter (Brea, CA, USA).

### Cell culture conditions

To analyze the expression of cytokines and transcription factors, the lymphocytes in NP tissues, uncinate tissues and PBMCs were stimulated for 5 h with PMA (20 ng/ml; Sigma-Aldrich) and ionomycin (1 μg/ml; Sigma-Aldrich) at 37 °C with 5% CO2 in the presence of brefeldin A (10 μg/ml; Sigma-Aldrich).

The lymphocytes in NP and uncinate tissues were stimulated with LPS (1 μg/ml; Sigma-Aldrich) and BFA for 8 h and the expression of IL-12 in CD14^+^ cells was detected by FACS.

The lymphocytes in NP tissues were stimulated with anti-CD3 (1 μg/ml) and anti-CD28 (1 μg/ml) in the presence or absence of IL-12 (5 ng/ml, eBioscience, Santiago, Chile) or IL-21 (50 ng/ml, Peprotech, Rocky Hill, NJ, USA) and anti-IL-12Rβ1 antibodies (50 ng/mL, Hoffmann-La Roche Inc, USA) for 3 days. Cell-free supernatants were harvested and assayed by ELISA for the assays of IL-21 or IFN-γ. The cells were collected, stimulated with PMA, ionomycin and BFA for 5 h, and the expression of IL-21 and IFN-γ was assayed by flow cytometry.

### ELISA

The lymphocytes in NP tissues and uncinate tissues were stimulated with LPS (1 μg/ml) for 24 h. The cell-free culture supernatants were harvested and assayed by ELISA for IL-12p40 production according to the manufacturer’s protocols.

The detection limits were as follows: IL-21 (eBioscience), 31 pg/mL; IFN-γ (BD Bioscience PharMingen), 4.7 pg/mL; and IL-12p70 (eBioscience), 0.16 pg/mL. For convenient analysis, all values of less than the detectable limit were considered zero.

### Immunofluorescence assay

Paraffin sections of NP tissues were rehydrated and boiled in EDTA buffer (pH8.0) for 10 min to induce antigen retrieval. After washing, tissue sections were blocked for nonspecific binding with 5% goat serum/0.3% Tween-20/PBS, and incubated with rabbit anti-human IL-21 antibody (polyclone, 1:100, abcam, Cambridge, MA, USA) or rabbit anti-human CXCR5 antibody (clone: EPR8837, 1:200, abcam) and mouse anti-CD4 antibody (clone: BC/1F6, 1:200, abcam) at 4 °C overnight. After washing, sections were incubated with Alexa Fluor 488–conjugated goat anti-mouse IgG (1:1000, Invitrogen, Carlsbad, CA, USA)) and Alexa Fluor 555–conjugated goat anti-rabbit IgG (1:1000, Invitrogen) for 2 hours at room temperature in the dark. After a final washing, cover slips were mounted onto slides with fluoroshield mounting medium with DAPI (4′,6-diamidino-2-phenylindole; abcam). The slides were observed with Olympus microscope BX53, and images were collected by using Cell Sense software (Olympus, Center Valley, Pa).

### Statistical analysis

Data are expressed as mean ± SEM or median (range). Comparison between two groups was performed by Student’s t-tests. Values of p < 0.05 (two tailed) was considered significant.

## Additional Information

**How to cite this article**: Xiao, L. *et al*. Human IL-21^+^IFN-γ^+^CD4^+^ T cells in nasal polyps are regulated by IL-12. *Sci. Rep.* **5**, 12781; doi: 10.1038/srep12781 (2015).

## Supplementary Material

Supplementary Information

## Figures and Tables

**Table 1:** Subjects’ characteristics.

characteristic	CRSwNP
Subject numbers	42
Age (y), mean (range)	43 (13 ~ 75)
Sex (male/female)	30/12
Duration (y), mean (range)	11.5 (1 ~ 40)
Atopic	7
Aspirin intolerance	0
CT score	15.5 (6 ~ 24)
Nasal endoscopy score	4.5 (1 ~ 6)
Mean VAS score	6.4 (3 ~ 8.1)
