# Supplementary material for: Human IL-21+IFN-γ+CD4+ T cells in nasal polyps are regulated by IL-12
Source: Sci Rep. 2015 Aug 4;5:12781. doi: 10.1038/srep12781 (PMC4523938; doi:10.1038/srep12781)
Supplement: Supplementary Information [file srep12781-s1.doc]

**Human IL-21^+^IFN-γ^+^CD4^+^ T cells in nasal polyps are regulated by IL-12**

**Authors**: Li Xiao^a^, Lei Jia^a^, Yannan Zhang^a^, Sifei Yu^a^, Xingmei Wu^b^, Binyan Yang^a^, Huabin Li^b^∗, Changyou Wu^a^∗

^a^ Institute of Immunology, Zhongshan School of Medicine, Key Laboratory of Tropical Disease Control Research of Ministry of Education, Sun Yat-sen University, Guangzhou, PR China

^b^ Allergy and Cancer Center, Otorhinolarygology Hospital, The First Affiliated Hospital of Sun Yat-sen University, Guangzhou, PR China

∗ **Corresponding author:**

Huabin Li, MD, Allergy and Cancer Center, Otorhinolarygology Hospital, The First Affiliated Hospital of Sun Yat-sen University, No. 58, Zhongshan 2nd Road, Guangzhou, China, 510080. Tel/Fax: 86 20 87333733; Email: allergyli@163.com. And Changyou Wu, MD, Ph D, Institute of Immunology, Zhongshan School of Medicine, Sun Yat-sen University, 74 Zhongshan 2nd Road, Guangzhou 510080, China. Tel/Fax: +86 20 87332448; E-mail address: changyou_wu@yahoo.com.

**Supplementary Figure 1:**


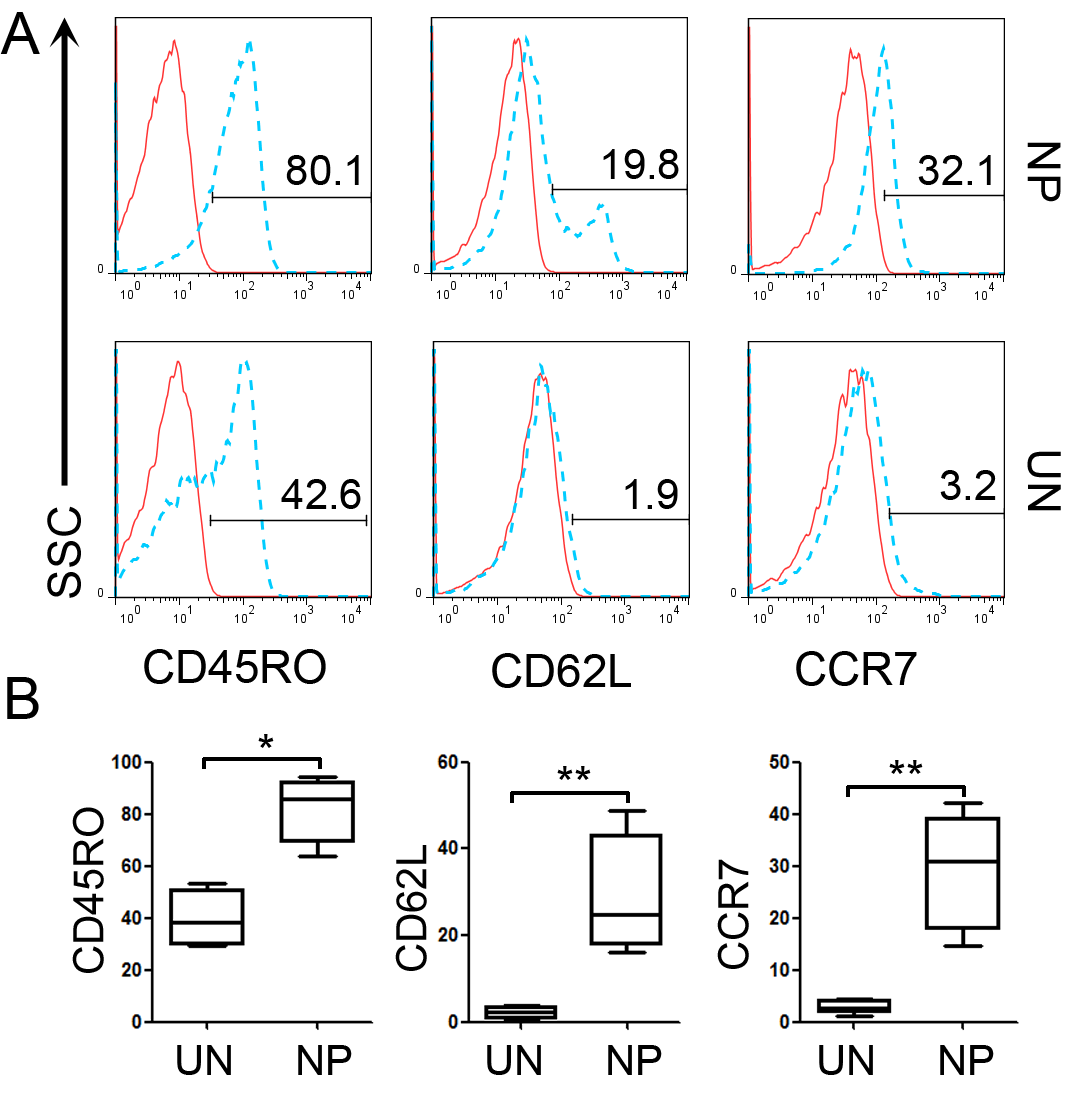


Supplementary Figure1: The expression of CD45RO, CD62L and CCR7 on CD4^+^ T cells in NP tissues was increased. (A, B) Representative data from FACS and statistical analysis showed the expression of CD45RO, CD62L and CCR7 on CD4^+^ T cells in NP and uncinate tissues without any stimulation (n=7). Data were shown as mean ± SEM. Statistical significance was determined with the Mann–Whitney test. * p<0.05; ** p<0.01. NP, nasal polyps; UN, uncinate.
